# Comprehensive detection of pathogens in immunocompromised children with bloodstream infections by next-generation sequencing

**DOI:** 10.1038/s41598-018-22133-y

**Published:** 2018-02-28

**Authors:** Kazuhiro Horiba, Jun-ichi Kawada, Yusuke Okuno, Nobuyuki Tetsuka, Takako Suzuki, Shotaro Ando, Yasuko Kamiya, Yuka Torii, Tetsuya Yagi, Yoshiyuki Takahashi, Yoshinori Ito

**Affiliations:** 1 Department of Pediatrics, Nagoya University Graduate School of Medicine, Nagoya, Japan; 2 Department of Infectious Disease, Nagoya University Hospital, Nagoya, Japan

## Abstract

Bloodstream infection (BSI) is a severe complication in immunocompromised patients. Next-generation sequencing (NGS) allows us to analyze comprehensively and quantitatively all microorganisms present in a clinical sample. Thirty-five pediatric patients (12 with BSI and 23 with suspected BSI/negative blood culture) were enrolled. Plasma/serum samples were used for sequencing and the results were compared with those from blood culture. Sequencing reads of bacteria isolated in blood culture were identified by NGS in all plasma/serum samples at disease onset. Bacteria isolated in blood culture were identical to the dominant bacteria by NGS in 8 of 12 patients. Bacterial reads per million reads of the sequence depth (BR) > 200 and relative importance values of the dominant bacteria (P1) > 0.5 were employed to determine causative pathogens. Causative pathogens were detected using these criteria in 7 of 12 patients with BSI. Additionally, causative bacteria were detected in the plasma/serum at 7 days before disease onset in two patients with catheter-related BSI. Causative pathogens, including virus, were identified in three patients with suspected BSI. Lastly, a total of 62 resistance genes were detected by NGS. In conclusion, NGS is a new method to identify causative microorganisms in BSI and may predict BSI in some patients.

## Introduction

Bloodstream infection (BSI) is a severe complication in immunocompromised patients, which can lead to sepsis in some cases. Due to the lack of rapid diagnostic procedures to identify causative microorganisms, initiation of empiric antibiotic therapy as early as possible is recommended^1^. BSI is often caused by bacteria and routinely diagnosed by blood culture, which is regarded as the gold standard diagnostic procedure. However, blood culture can only detect culturable pathogens, and less than 30% of blood cultures from febrile neutropenia and 50% from septic shock are reported to be positive^2–4^. Prompt identification of causative microorganisms would improve the outcome of BSI due to optimization of antimicrobial treatment. In this context, culture-independent molecular diagnostic procedures, such as PCR-based approaches, have been developed^5,6^. However, further efforts appear to be needed to establish rapid and comprehensive diagnostic procedures.

Next-generation sequencing (NGS) is a culture-free method that can analyze the entire microbial community within a sample. Shotgun metagenomic sequencing allows us to obtain comprehensively and quantitatively all genes in all organisms present in a clinical sample. Several reports have attempted to identify pathogens in infectious diseases, such as febrile illness^7^, respiratory and gastric/digestive infection^8,9^, and acute encephalitis/encephalopathy^10^. Additionally, some studies investigated causative microorganisms in blood samples from patients with BSI using NGS^11–13^. However, there are currently no established methods to identify causative microorganisms of BSI by NGS, and few studies have examined this method in pediatric patients^7^. Therefore, the present study was conducted to establish an NGS-based method to diagnose BSI in pediatric patients.

## Results

### Sequencing reads by NGS

We analyzed 37 plasma/serum samples from the BSI group and 23 samples from the suspected BSI group by NGS. We obtained an average of 18.3 million reads per sample. While most of the reads were attributed to human-derived DNA and artifacts, 0.09% of the total reads were derived from bacteria with high confidence (defined by bit score >250).

### NGS results at the onset of BSI in comparison with blood culture results

**Figure 1:**
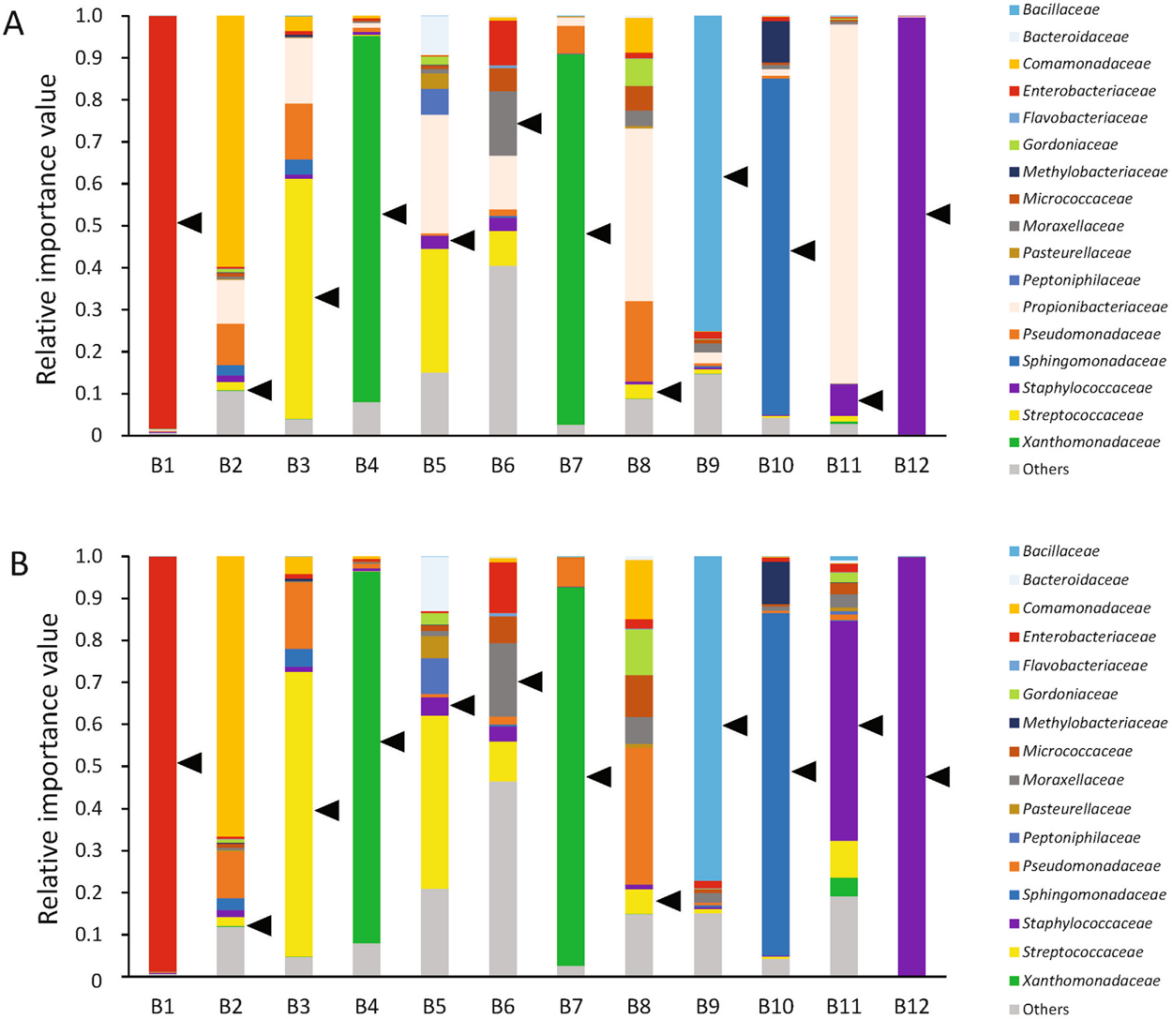
Relative importance value of bacteria in patients with bloodstream infection Each bar represents taxa at the family level of taxonomic hierarchy. Arrowheads indicate bacteria isolated by blood culture. (**A**) Relative importance values including reads of *Propionibacterium*. (**B**) Relative importance values after removal of reads of *Propionibacterium*.

Relative importance values of detected bacterial reads from plasma/serum samples at the onset of BSI in the BSI group are demonstrated in Fig. 1A. The reads classified as *P*. *acnes* were discarded before further analysis, as shown in Fig. 1B and Supplementary Figure 1, because *P*. *acnes* is considered normal bacterial flora of human skin. The sequencing reads of bacteria isolated in blood culture were identified by NGS at the family level of taxonomic hierarchy in all plasma/serum samples at the onset of BSI. Additionally, bacteria isolated in blood culture were identical to the “dominant” bacteria by NGS in 8 of 12 patients. The detected bacteria were identical to the dominant bacteria at the species level in patients B1, B3, B4, B7, and B12; at the genus level in patients B9 and B11; and at the family level in patient B10 (Table 1). In patients B2 and B11, sequencing reads relevant to *Capnocytophaga sputigena* or *Staphylococcus lugdunensis*, which was identified in blood culture, could not be identified up to the family level because the number of sequencing reads was small.

**Table 1:** Summary of next-generation sequencing results at the onset of bloodstream infection.

Patient ID	Sequence depth (reads)	Bacteria isolated from blood culture	Reads (per million reads)^*^	Dominant bacteria by NGS (species)**	Reads (per million reads) ^*^
B1	9,895,348	*Escherichia coli*	2,575	*Escherichia coli*	2,575
B2	9,753,018	*Capnocytophaga sputigena*	0.3	*Delftia* sp. strain Cs1-4	179
B3	21,983,948	*Streptococcus oralis/mitis*	444/6	*Streptococcus mitis*	444
B4	32,895,328	*Stenotrophomonas maltophilia*	267	*Stenotrophomonas maltophilia*	267
B5	10,166,160	*Staphylococcus aureus*	7	*Streptococcus pyogenes*	223
B6	22,769,288	*Acinetobacter ursingii* ^†^	1	*Moraxella osloensis*	4
B7	16,968,966	*Stenotrophomonas maltophilia*	1,415	*Stenotrophomonas maltophilia*	1,415
B8	4,793,796	*Streptococcus oralis/mitis*	7/3	*Pseudomonas stutzeri*	172
B9	24,026,704	*Bacillus cereus*	54	*Bacillus anthracis*	80
B10	20,027,612	*Sphingomonas paucimobilis*	25	*Sphingobium* sp. strain TKS	34
B11	9,305,716	*Staphylococcus lugdunensis* ^†^	36	*Staphylococcus epidermidis*	25
B12	17,397,938	*Staphylococcus epidermidis*	27,719	*Staphylococcus epidermidis*	27,719

^*^The number of the reads derived from isolated bacteria in blood culture per million reads of sequencing.

**The reads classified as *Propionibacterium acnes* were discarded.

^†^These bacteria were classified at the genus level.

### Evaluation of BR and P1 indices to diagnose causative bacteria

BR was calculated as the bacterial reads per million reads of the sequence depth and P1 was calculated as the percentages of the reads of the dominant bacteria. To determine the clinical values using BR and P1 indices, NGS results were analyzed. First, BR of 8 patient samples, in which the dominant bacteria by NGS were consistent with the results of blood culture both at the onset and post-onset of BSI, was plotted (Supplementary Figure 2A). The post-onset group, in which the causative bacteria significantly decreased at post-onset of the disease, was postulated as the control group. Because a BR value of 200 appeared to be able to discriminate between the onset group and the post-onset group, BR > 200 was adopted as the threshold value. Second, P1 at the family level in 8 patients (Supplementary Figure 2B), P1 at the genus level in 7 patients (Supplementary Figure 2C), and P1 at the species level in 5 patients (Supplementary Figure 2D) were plotted both at the onset and post-onset of BSI. The post-onset group was again postulated as the control group. Because a P1 value of 0.5 appeared to be able to discriminate between the onset group and the post-onset group, P1 > 0.5 was adopted as the threshold value. Using these indices, 7 of 8 patient samples, in which the dominant bacteria by NGS were consistent with the results of blood culture at the onset of BSI, fulfilled the criteria of BR > 200 and P1 > 0.5 (Table 2). These seven samples were considered as NGS positive in this study. In addition, 8–94% sequence coverage of the total genome of the representative strain of bacteria detected in blood culture was obtained in these seven samples (Table 2, Supplementary Figure 3).

**Table 2:** Identification of causative bacteria by next-generation sequencing in the bloodstream infection group.

Patient	Bacterial reads per million reads of the sequence depth (BR)	Relative importance value of the dominant bacteria (P1)			Causative bacteria by next-generation sequencing^*^ (taxonomic hierarchy)	Comparison to blood culture	Coverage of the reference sequence
		Family	Genus	Species			
B1	2,634	0.99	0.98	0.98	*Escherichia coli* (species)	identical	0.37
B2	489	0.68	0.67	0.37	*Comamonadaceae* (family)	different	—
B3	991	0.68	0.68	0.45	*Streptococcus* (genus)	identical	0.61
B4	305	0.90	0.89	0.88	*Stenotrophomonas maltophilia* (species)	identical	0.49
B5	1,129	0.43	0.43	0.20	Not applicable	—	—
B6	25	0.21	0.18	0.18	Not applicable	—	—
B7	1,614	0.90	0.89	0.88	*Stenotrophomonas maltophilia* (species)	identical	0.62
B8	639	0.33	0.33	0.27	Not applicable	—	—
B9	200	0.83	0.83	0.40	*Bacillus* (genus)	identical	0.08
B10	1,839	0.82	0.42	0.19	*Sphingomonadaceae* (family)	identical	0.38
B11	70	0.52	0.52	0.36	Not applicable	—	—
B12	29,330	1.00	1.00	0.95	*Staphylococcus epidermidis* (species)	identical	0.94

^*^Causative bacteria fulfilled the criteria of BR > 200 and P1 > 0.50. The lowest taxonomic hierarchy which met P1 > 0.50 was applied.

### Evaluation of BR/P1/H’ indices in consecutive samples pre- and post-onset of BSI

**Figure 2:**
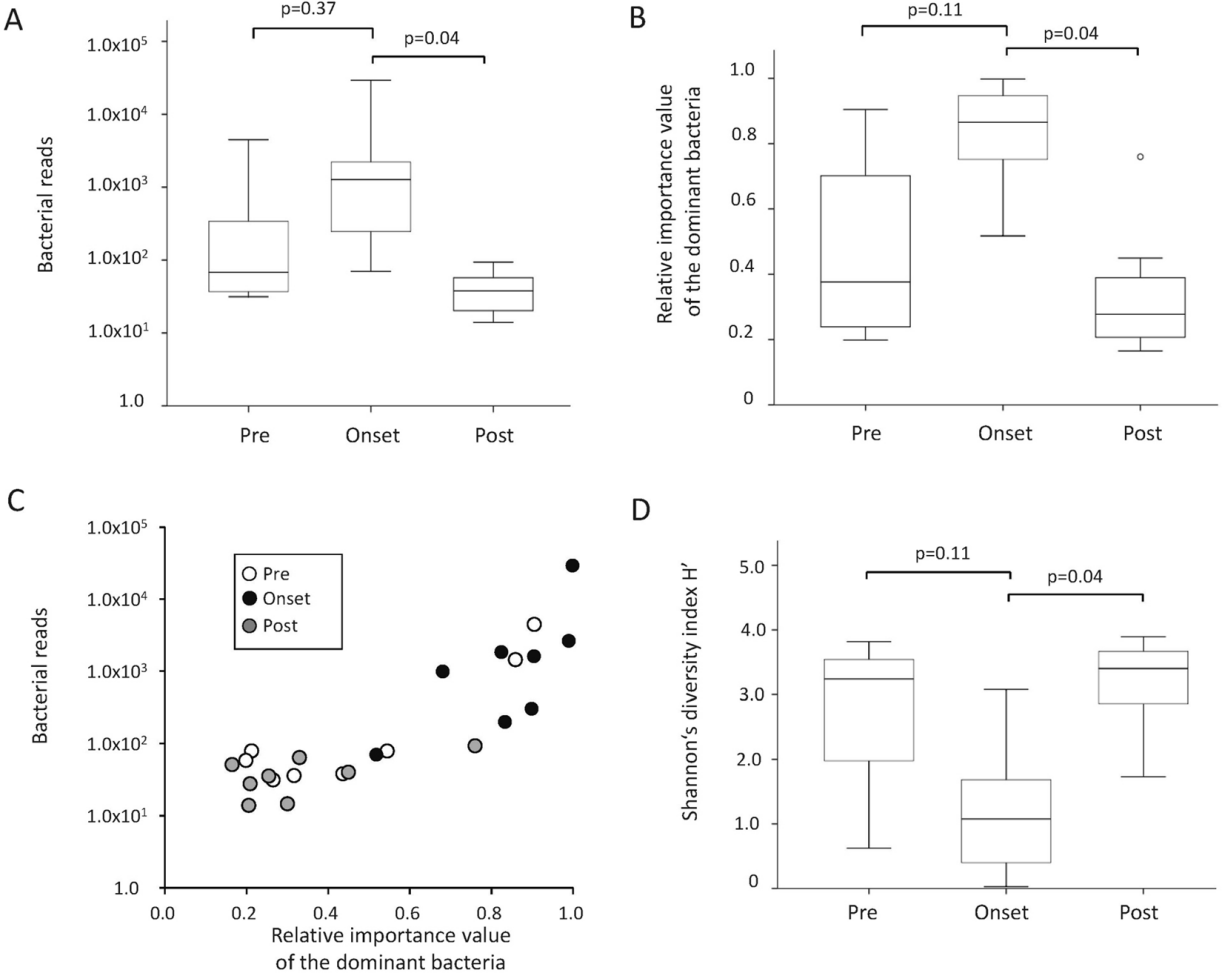
Index values in consecutive samples in bloodstream infection patients in whom the dominant bacteria by next-generation sequencing was identical to bacteria isolated in blood culture. (**A**) Bacterial reads per million reads of sequencing (BR) are demonstrated at consecutive time points. (**B**) Relative importance value of the dominant bacteria (P1) is shown at consecutive time points. (**C**) Score plots of BR and P1. Samples at onset (closed circles) clustered separately from those at post-onset (grey circles). (**D**) Shannon’s diversity index at the family level (H’) is shown at consecutive time points.

To evaluate BR and P1 as diagnostic indices, samples from the eight patients with identical causative bacteria by NGS and blood culture were analyzed at consecutive time points of BSI. BR values were significantly higher in samples at onset than in those at post-onset (Fig. 2A, p = 0.04). P1 values at the family level were also significantly higher in samples at onset than in those at post-onset (Fig. 2B, p = 0.04). However, P1 values at the genus or species level were not significantly different between consecutive time points (p = 0.05 in genus and p = 0.20 in species).

Samples at onset appeared to be clustered in comparison to samples at pre/post-onset, although some samples at pre-onset did not discriminate from samples at onset (Fig. 2C). Additionally, H’ values were significantly different between samples at onset and those at post-onset (p = 0.04) (Fig. 2D).

### NGS analysis of samples at pre-onset

**Figure 3:**
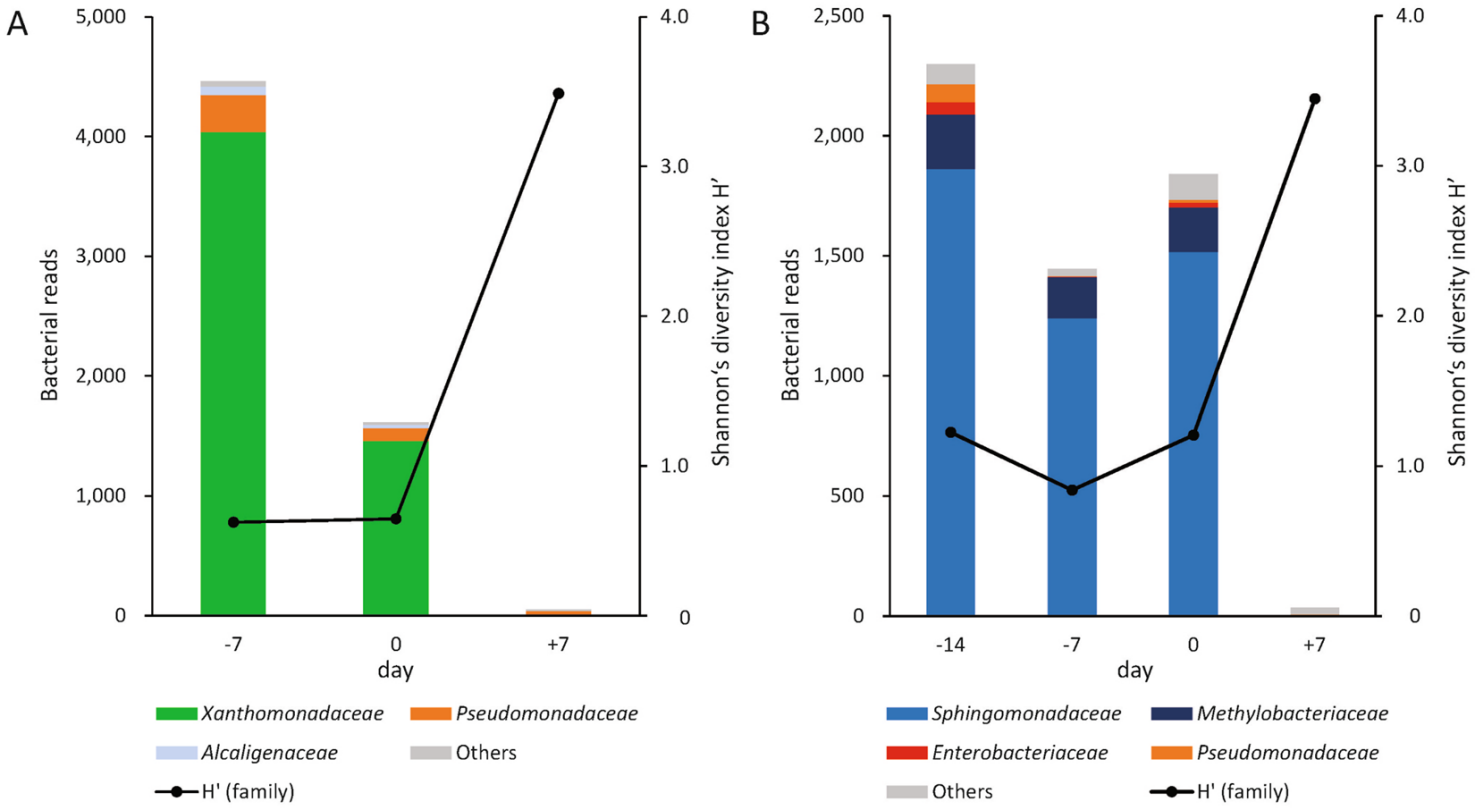
Time course analyses by next-generation sequencing for two patients with catheter-related bloodstream infection. (**A**) Time course of patient B7. *Stenotrophomonas maltophilia* was cultured from his blood at the onset of bloodstream infection (BSI). Patient B7 was a 10-year-old boy who received umbilical cord blood transplantation to treat neuroblastoma. He developed fever and chills and BSI was suspected. Immediately after symptom onset, he was treated with cefozopran, a cephalosporin antibiotic. Ceftazidime was added because the fever persisted after cefozopran treatment. Finally, the central venous catheter was removed 4 days after onset and *S*. *maltophilia* was cultured from the catheter tip. The patient recovered after catheter removal. (**B**) Time course of patient B10. *Sphingomonas paucimobilis* was cultured from his blood. Patient B10 was an 8-year-old boy who received umbilical cord blood transplantation to treat neuroblastoma. He developed a fever and BSI was suspected. Immediately after fever onset, he was treated with cefozopran. Finally, the central venous catheter was removed 4 days after onset and *S*. *paucimobilis* was cultured from the catheter tip. The patient recovered after catheter removal.

Four samples (2 from patient B2, 1 from patient B7, and 1 from patient B10) at pre- and post-onset met the criteria of BR > 200 and P1 > 0.5. The NGS results of patients B7 and B10 are shown in Fig. 3. Interestingly, NGS analysis in patient B10 was performed for the sample at 2 weeks pre-onset. The NGS result was positive for *Sphingomonadaceae*, which was identical to the results at disease onset. Patients B7 and B10 were diagnosed with catheter-related BSI (CRBSI) by culture methods because of bacteremia originating from an intravenous catheter. Concerning patient B2, *Delftia* identified at onset was identified from samples at pre- and post-onset by NGS. However, this result was not consistent with the bacterium isolated in blood culture (*C*. *sputigena*).

### Evaluation of samples in the suspected BSI group using BR and P1 indices

**Figure 4:**
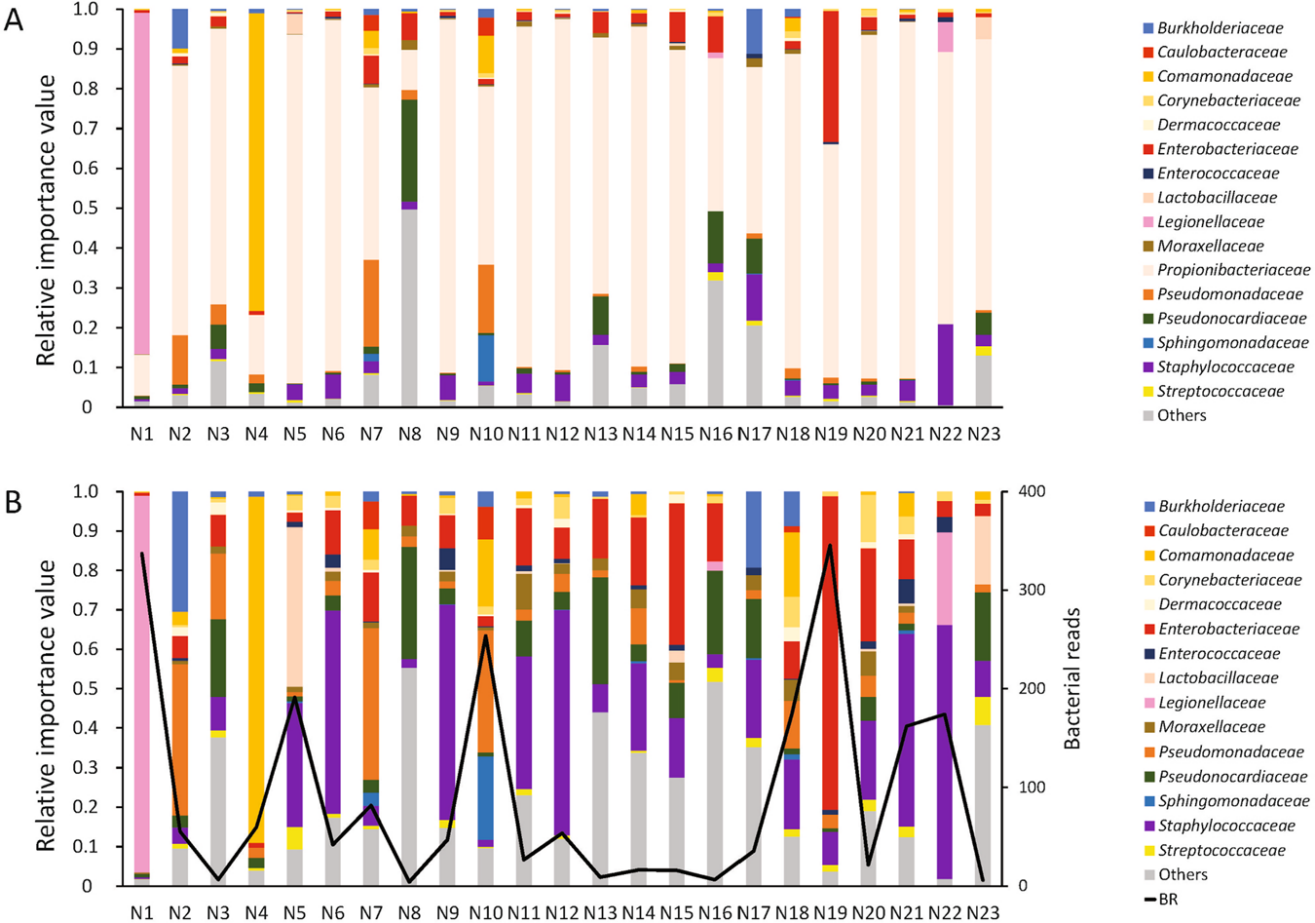
Relative importance value of bacteria in patients with suspected bloodstream infection. Each bar represents taxa at the family level of taxonomic hierarchy. (**A**) Relative importance values including reads of *Propionibacterium*. (**B**) Relative importance values after removal of reads of *Propionibacterium*. *Tatlockia micdadei* (*Legionellaceae*) in patient N1 and *Escherichia coli* (*Enterobacteriaceae*) in patient N19 were considered the causative bacteria. In patient N22, *human mastadenovirus* had the highest number of reads (data not shown). The black line shows bacterial reads per million reads of the sequence depth.

Numerous bacteria reads were detected by NGS in 23 plasma/serum samples from suspected BSI patients (Fig. 4). Of these, patients N1 and N19 fulfilled the criteria of BR > 200 and P1 > 0.5 (Table 3). Thirty-one percent of the total genome of the representative strain of *Tatlockia micdadei* (genus *Legionella*) was sequenced in patient N1 (Supplementary Figure 4A). A specific region of *Legionella* species was amplified using PCR (Supplementary Figure 5A). Patient N19 was diagnosed as having BSI caused by *E*. *coli*. The reads of various regions of the *E*. *coli* genome were detected. However,<1% of the total genome was covered (Supplementary Figure 4B) and the uid A region of *E*. *coli* was not amplified by PCR. Patient N22 was diagnosed as having BSI caused by *human mastadenovirus* based on NGS data after data processing without analysis of BR and P1 values. The *human mastadenoviru*s-derived reads completely covered the human adenovirus 2 reference genome (Supplementary Figure 4C). The presence of human adenovirus in the serum of patient N22 was confirmed by PCR (Supplementary Figure 5B).

**Table 3:** Identification of causative bacteria by next-generation sequencing in the suspected bloodstream infection group.

Patient ID	Sequence depth (reads)	Bacterial reads per million reads of the sequence depth (BR)	Total viral reads^*^	Relative importance value of the dominant bacteria (P1)			Microorganisms detected by next-generation sequencing^**^ (taxonomic hierarchy)	Coverage of the reference sequence
				Family	Genus	Species		
N1	22,430,302	337	1	0.97	0.97	0.97	*Tatlockia micdadei* (species)	0.31
N2	25,372,464	55	4	0.41	0.41	0.23	Not applicable	—
N3	22,509,896	6	2	0.30	0.30	0.30	Not applicable	—
N4	8,166,236	60	2	0.90	0.89	0.49	Not applicable	—
N5	33,363,292	191	13	0.39	0.39	0.37	Not applicable	—
N6	30,209,696	42	4	0.53	0.53	0.31	Not applicable	—
N7	21,437,914	82	0	0.41	0.41	0.32	Not applicable	—
N8	21,055,106	4	0	0.62	0.62	0.62	Not applicable	—
N9	30,648,150	47	3	0.56	0.56	0.29	Not applicable	—
N10	20,770,680	254	1	0.32	0.32	0.25	Not applicable	—
N11	30,840,058	27	7	0.40	0.40	0.17	Not applicable	—
N12	23,245,940	54	16	0.57	0.57	0.42	Not applicable	—
N13	11,217,618	9	51	0.45	0.45	0.45	Not applicable	—
N14	10,154,076	16	3	0.25	0.25	0.18	Not applicable	—
N15	7,134,884	16	4	0.43	0.24	0.24	Not applicable	—
N16	14,575,648	6	0	0.39	0.39	0.39	Not applicable	—
N17	11,089,400	35	1	0.25	0.25	0.20	Not applicable	—
N18	20,458,358	174	3	0.18	0.18	0.09	Not applicable	—
N19	7,358,930	346	9	0.81	0.80	0.80	*Escherichia coli* (species)	< 0.01
N20	32,804,676	22	2	0.26	0.23	0.23	Not applicable	—
N21	25,507,124	162	16	0.44	0.44	0.31	Not applicable	—
N22	28,784,934	174	20,727	0.60	0.60	0.38	*Human mastadenovirus* (species)	1.00
N23	10,458,628	6	0	0.27	0.27	0.27	Not applicable	—

^*^The number of reads derived from viruses per million reads of sequencing.

^**^Causative bacteria fulfilled the criteria of BR > 200 and P1 > 0.50. The lowest taxonomic hierarchy which met P1 > 0.50 was applied.

### Identification of antimicrobial resistance genes

Antimicrobial resistance genes were investigated using NGS data in seven patients in the BSI group and two patients in the suspected BSI group. A total of 62 resistance genes were found in 1,411 registered gene sequences in the database. The obtained sequence data of these genes covered 5–100% of reference sequences throughout the whole region of each gene. Resistance genes covering >50% of reference sequences are shown in Supplementary Table 3.

## Discussion

NGS analyses with specific indices were proposed in this study to develop a comprehensive diagnostic method for infectious diseases. BR represents the number of bacteria-derived reads sequenced from blood samples. As expected, BR indicated an apparent increase in the number of bacterial genomes and was larger at the onset of BSI *vs* pre- or post-onset in most cases. We also introduced P1 as an index for specifying causative bacteria and considered detected bacterial reads as significant if the dominant bacterial reads accounted for more than half of the total bacterial reads. Among patients with BSI, significant bacterial reads were detected at the family level in 8 patients (Table 2).

To our knowledge, there has been no report on NGS analysis of samples before the onset of BSI. Interestingly, substantial reads of bacteria identical to those isolated in blood culture at fever onset were detected 7 or 14 days before onset in two patients with CRBSI (patients B7 and B10), suggesting that the causative pathogen had colonized the patient >7 days before the onset of fever. The blood may harbor dormant microorganisms or microorganisms may enter the bloodstream due to translocation or pathological conditions^14^. Bacteria isolated in blood culture at the onset of BSI were identified by NGS from pre-onset samples, which may be attributed to blood collection via central venous catheter. Thus, an NGS-based approach may help to predict the development of CRBSI.

Not all bacteria isolated by blood culture were identical to the dominant bacteria detected by NGS in patients with BSI. It is possible blood culture contaminants were included in these discrepant samples because only one bottle of blood was cultured in most patients in the present study. *C*. *sputigena*, *Acinetobacter ursingii*, and *S*. *lugdunensis* were isolated in three patients with discrepant results. In general, these bacteria rarely cause BSI, suggesting the possibility of blood culture contamination^15–17^. *P*. *acnes* is the most abundant bacterium on human skin^18^, therefore, we first removed the reads of *P*. *acnes* from the NGS results in this study. However, removing only the reads of *P*. *acnes* may not sufficiently address contamination of skin microbiome because skin flora can vary per individual. Additionally, in patient B2, *Delftia* species was identified at all time points despite improved symptoms. *Delftia* is a common water and soil saprophyte that is usually considered a nonpathogenic organism in the clinical setting^19^. Therefore, metagenomic analysis of skin flora before examining blood samples is necessary to consider more precisely contamination of skin flora.

Causative bacteria and virus were identified in 3 of 23 patients in the suspected BSI group. Patient N1 had had a central venous catheter and there was an opportunity for *T*. *micdadei* to cause BSI because *T*. *micdadei* is an environmental bacteria^20^. Immediately after fever, the patient was treated with pazufloxacin, a fluoroquinolone antibiotic, which is generally effective against *Legionella* species, and recovered. Patient N19, who received umbilical cord blood transplantation, had been treated with panipenem/betamipron for 4 days due to fever. He recovered after the addition of arbekacin, which is generally effective against *E*. *coli*. Adenovirus was identified in patient N22, who showed hematuria and hepatic dysfunction.

Patients in the BSI group with discrepant results between blood culture and NGS analysis may not have apparent causative agents in blood. There may be other reasons for fever or elevation of CRP than infection in these patients^21^. Patient B6 had a prominent rise in white blood cell count before fever onset after receiving hematopoietic stem cell transplantation. Laboratory findings on the next day confirmed recurrence of acute lymphoid leukemia. Negative NGS results may also indicate that inflammatory reactions in patients are not caused by infectious diseases because NGS is a comprehensive and quantitative diagnostic procedure with high sensitivity. From this viewpoint, diagnosis by NGS can possibly avoid antimicrobial overtreatment or antibiotic-related toxicity and decrease multidrug-resistant pathogens due to the use of broad-spectrum antibiotics.

Previous reports have shown the potential of NGS-based methods to identify genes conferring antibiotic resistance in plasma of BSI patients^12,13^. Tetracycline, macrolide-lincosamide-streptogramin B, methicillin, and vancomycin resistance genes were found. Because antimicrobial resistance genes can be simultaneously detected with identification of causative microorganisms, NGS analysis is advantageous for selecting antimicrobial treatment. In the present study, a total of 62 resistance genes were detected in nine patients with sequences covering 5–100% of reference sequences. However, obtained sequences of two-thirds of these genes were considered insufficient. Insufficient coverage of reference sequences was due to the relatively low quality of DNA from stored samples and a large amount of human-derived DNA. Further studies are needed to fully obtain the sequencing data of these resistance genes.

Data processing is just as critical as sequencing in the workflow for identifying causative microorganisms by NGS. Currently, different cloud-computing pipelines and public databases are applied to process raw data from sequencing^22–24^. However, the database of known sequences is insufficient for identifying causative microorganisms at the species level. For bacteria such as *Bacillus cereus*, it might be difficult to discriminate lower levels of taxonomic hierarchy by sequencing of 150 base fragments because of high genomic homology among species^25^. Moreover, *Sphingomonas paucimobilis*, the causative bacteria of BSI in patient B10, has only a draft genome as the reference genome and it was difficult to classify correctly the bacterium from fragmented reads. Additionally, because the number of resistance genes has increased, databases should be continuously updated.

In conclusion, the present study demonstrates the advantage of NGS analysis for identifying causative pathogens in patients with diagnosed or suspected BSI. Causative bacteria that were not isolated in blood culture and viruses could be detected by using NGS. Additionally, NGS could identify antimicrobial resistance genes. Although routine use of NGS may be difficult because it is costly and labor intensive, an NGS-based approach has great potential to detect causative pathogens of infectious diseases.

## Methods

### Ethics Statement

The study design and methods were approved by the Institutional Review Board of Nagoya University Hospital (no. 9069). The methods were carried out in accordance with the approved guidelines. Written informed consent was obtained from all patients or their guardians.

### Patients and samples

Thirty-five pediatric patients, treated between June 2010 and August 2016, were enrolled in this study. BSI was diagnosed using the following criteria: 1) pathogen detected from blood culture and 2) fever ≥ 38.0 °C (axillary temperature) or elevated C-reactive protein (CRP) (>1.0 mg/dL). Twelve pediatric patients with BSI were defined as the BSI group and 23 pediatric patients with suspected BSI and negative blood cultures were defined as the suspected BSI group. Clinical characteristics of the patients are shown in Supplementary Tables 1 and 2. All 12 patients with BSI and 21 of 23 patients with suspected BSI were immunocompromised after treatment for cancer and had intravascular catheters. Two patients with suspected BSI had sepsis. When we applied the criteria for systemic inflammatory response syndrome proposed by a previous study^26^, 9 of 12 patients in the BSI group and 21 of 23 patients in the suspected BSI group met these criteria.

At least 1 mL of blood were collected from each patient for culture either at fever onset or CRP elevation before intravenous broad-spectrum antibiotic and/or antifungal treatment was initiated. In the BSI group, 37 plasma/serum samples (28 plasma and 9 serum samples) collected at consecutive time points were used for NGS: 1) ≥ 3 days pre-onset of BSI, 2) 24 h pre- and 24 h post-onset of BSI, and 3) ≥ 3 days post-onset of BSI. In the suspected BSI group, 23 plasma/serum samples (13 plasma and 10 serum samples) collected 24 h pre- and 24 h post-onset of BSI were used for NGS. Collected plasma/serum samples were stored at −30 °C until NGS analysis.

### Blood culture

One bottle of BD BACTEC^TM^ Peds Plus^TM^/F Culture Vials (Becton Dickinson and Company, Franklin Lakes, NJ, USA) was used for each patient. Whole blood samples from all 35 patients were analyzed using the blood culture instrument BD BACTEC^TM^ FX (Becton Dickinson and Company) according to the manufacturer’s instructions. Positive blood culture bottles were identified using the automated microbial identification system VITEK MS system (bioMérieux, Lyon, France).

### Sample preparation for NGS and sequencing

Plasma/serum samples were filtered through a 5.0-μm filter (Merck Millipore, Temecula, CA, USA) to remove blood cells. DNA was extracted using the QIAamp UCP Pathogen Mini Kit (Qiagen, Hilden, Germany) and measured using a Qubit dsDNA BR Assay Kit (Thermo Fisher Scientific, Waltham, MA, USA). DNA sequencing libraries were prepared using a Nextera XT library Prep Kit (Illumina, San Diego, CA, USA) according to the manufacturer’s instructions with slight modification: 14 cycles of PCR for amplification of tagmented DNA was carried out and the final elution was carried out in 42.5 μL of resuspension buffer after the bead clean-up step (Illumina). Library quality was determined using an Agilent 2200 TapeStation (Agilent, Santa Clara, CA, USA). Libraries were then sequenced on a HiSeq2500 (Illumina) with the 2 × 150 bp pair-end protocol.

### NGS data processing

NGS data were processed using the cloud-computing pipeline for metagenomic identifying pathogens, MePIC v2.0 (Pathogen Genomics Center, National Institute of Infectious Diseases, Japan) as follows^23^. Adaptor sequences and low-quality bases were trimmed from raw sequencing reads, followed by subtraction of human-derived reads. Subsequently, the remaining sequence reads were mapped against known nucleotide sequences in the database including microbial genomes using MEGABLAST programs. To summarize taxonomic information, the metagenomic browser MEGAN6 Community Edition (University of Tübingen, Tübingen, Germany) was used^27^. After low accuracy reads (bit score <250), unclassified reads, and Illumina sequencing spike-in PhiX reads were excluded, “bacterial reads” were obtained. The reads classified as *Propionibacterium acnes* were discarded as contaminants in downstream analysis. Moreover, index values were evaluated for diagnostic purpose. Bacterial reads per million reads of the sequence depth (BR) were calculated in each plasma/serum sample. Relative importance values of the dominant bacteria (P1) were also calculated as percentages of the reads of the “dominant” bacteria, which had the highest number of reads. Additionally, Shannon’s diversity index (H’) based on each taxonomic hierarchy was calculated from the bacterial reads and analyzed in consecutive samples from the same patient. Alignment with the reference genome of each microbe was visualized by CLC genomics workbench 9.0 (CLC bio, Waltham, MA, USA). The detected sequences were grouped into a specific species, however, genus or family was exclusively determined in several samples. The ResFinder database^28^ was used to detect antimicrobial resistance genes.

### Validation by PCR

The dominant bacteria and virus detected by NGS in patients N1, N19, and N22 were validated by PCR. *Legionella* species in patient N1, *Escherichia coli* in patient N19, and adenovirus in patient N22 were detected according to methods described previously^29–31^.

### Statistical analysis

Three index values, BR, P1, and H’, at three time points were analyzed by the Friedman test, and post hoc analysis was conducted using the Wilcoxon signed-rank test with Bonferroni correction. All statistical analyses were performed using SPSS version 24.0 (IBM, Chicago, IL, USA). P values < 0.05 were considered statistically significant.

## Electronic supplementary material

Supplementary information

## References

[1] Freifeld AG (2011). Clinical practice guideline for the use of antimicrobial agents in neutropenic patients with cancer: 2010 update by the infectious diseases society of america. Clin Infect Dis..

[2] Ramphal R (2004). Changes in the etiology of bacteremia in febrile neutropenic patients and the susceptibilities of the currently isolated pathogens. Clin Infect Dis..

[3] Hamalainen S (2008). Neutropenic fever and severe sepsis in adult acute myeloid leukemia (AML) patients receiving intensive chemotherapy: Causes and consequences. Leuk Lymphoma.

[4] Rosenberg PS (2006). The incidence of leukemia and mortality from sepsis in patients with severe congenital neutropenia receiving long-term G-CSF therapy. Blood.

[5] Su G (2015). 16S Ribosomal Ribonucleic Acid Gene Polymerase Chain Reaction in the Diagnosis of Bloodstream Infections: A Systematic Review and Meta-Analysis. PloS one.

[6] Liesenfeld O, Lehman L, Hunfeld KP, Kost G (2014). Molecular diagnosis of sepsis: New aspects and recent developments. Eur J Microbiol Immunol..

[7] Wylie KM, Mihindukulasuriya KA, Sodergren E, Weinstock GM, Storch GA (2012). Sequence analysis of the human virome in febrile and afebrile children. PloS one.

[8] Mizrahi H (2017). Comparison of sputum microbiome of legionellosis-associated patients and other pneumonia patients: indications for polybacterial infections. Sci Rep..

[9] Joensen KG (2017). Evaluating next-generation sequencing for direct clinical diagnostics in diarrhoeal disease. Eur J Clin Microbiol Infect Dis..

[10] Kawada J (2016). Identification of Viruses in Cases of Pediatric Acute Encephalitis and Encephalopathy Using Next-Generation Sequencing. Sci Rep..

[11] Long Y (2016). Diagnosis of Sepsis with Cell-free DNA by Next-Generation Sequencing Technology in ICU Patients. Arch Med Res..

[12] Gyarmati P (2016). Metagenomic analysis of bloodstream infections in patients with acute leukemia and therapy-induced neutropenia. Sci Rep..

[13] Grumaz S (2016). Next-generation sequencing diagnostics of bacteremia in septic patients. Genome Med..

[14] Potgieter M, Bester J, Kell DB, Pretorius E (2015). The dormant blood microbiome in chronic, inflammatory diseases. FEMS Microbiol Rev..

[15] Loubinoux J (2003). Bacteremia caused by Acinetobacter ursingii. J Clin Microbiol..

[16] Choi SH (2010). Incidence, characteristics, and outcomes of Staphylococcus lugdunensis bacteremia. J Clin Microbiol..

[17] Kim JA, Hong SK, Kim EC (2014). Capnocytophaga sputigena bacteremia in a patient with chronic lymphocytic leukemia. Ann Lab Med..

[18] Mollerup S (2016). Propionibacterium acnes: Disease-Causing Agent or Common Contaminant? Detection in Diverse Patient Samples by Next-Generation Sequencing. J Clin Microbiol..

[19] Chotikanatis K, Backer M, Rosas-Garcia G, Hammerschlag MR (2011). Recurrent intravascular-catheter-related bacteremia caused by Delftia acidovorans in a hemodialysis patient. J Clin Microbiol..

[20] Muder RR, Yu VL (2002). Infection due to Legionella species other than L. pneumophila. Clin Infect Dis..

[21] Toussaint E (2006). Causes of fever in cancer patients (prospective study over 477 episodes). Support Care Cancer..

[22] Kostic AD (2011). PathSeq: software to identify or discover microbes by deep sequencing of human tissue. Nat Biotechnol..

[23] Takeuchi F (2014). MePIC, metagenomic pathogen identification for clinical specimens. Jap J Infect Dis..

[24] Naccache SN (2014). A cloud-compatible bioinformatics pipeline for ultrarapid pathogen identification from next-generation sequencing of clinical samples. Genome Res..

[25] Rasko DA, Altherr MR, Han CS, Ravel J (2005). Genomics of the Bacillus cereus group of organisms. FEMS Microbiol Rev..

[26] Goldstein B, Giroir B, Randolph A (2005). International pediatric sepsis consensus conference: definitions for sepsis and organ dysfunction in pediatrics. Pediatr Crit Care Med..

[27] Huson DH (2016). MEGAN Community Edition - Interactive Exploration and Analysis of Large-Scale Microbiome Sequencing Data. PLoS Comput Biol.

[28] Zankari E (2012). Identification of acquired antimicrobial resistance genes. J Antimicrob Chemother..

[29] Yamamoto H, Hashimoto Y, Ezaki T (1993). Comparison of detection methods for Legionella species in environmental water by colony isolation, fluorescent antibody staining, and polymerase chain reaction. Microbiol Immunol..

[30] Watanabe M (2005). Detection of adenovirus DNA in clinical samples by SYBR Green real-time polymerase chain reaction assay. Pediatr Int..

[31] Frahm E, Obst U (2003). Application of the fluorogenic probe technique (TaqMan PCR) to the detection of Enterococcus spp. and Escherichia coli in water samples. J Microbiol Methods..

