# Supplementary material for: Comprehensive detection of pathogens in immunocompromised children with bloodstream infections by next-generation sequencing
Source: Sci Rep. 2018 Feb 28;8:3784. doi: 10.1038/s41598-018-22133-y (PMC5830625; doi:10.1038/s41598-018-22133-y)
Supplement: Supplementary file 1 — Supplementary information [file 41598_2018_22133_MOESM1_ESM.pdf]

Supplementary Information

Comprehensive detection of pathogens in immunocompromised children with bloodstream infections by next-generation sequencing

Kazuhiro Horiba[1], Jun-ichi Kawada[1], Yusuke Okuno[1], Nobuyuki Tetsuka[2], Takako Suzuki[1], Shotaro Ando[1], Yasuko Kamiya[1], Yuka Torii[1], Tetsuya Yagi[2], Yoshiyuki Takahashi[1], and Yoshinori Ito[1]*

[1]Department of Pediatrics, Nagoya University Graduate School of Medicine, Nagoya, Japan

[2]Department of Infectious Disease, Nagoya University Hospital, Nagoya, Japan

***Corresponding author:** Yoshinori Ito, M.D., Ph.D.

Department of Pediatrics, Nagoya University Graduate School of Medicine

65 Tsurumai-cho, Showa-ku, Nagoya 466-8550, Japan

Tel: +81-52-744-2294; Fax: +81-52-744-2974

E-mail: yoshi-i@med.nagoya-u.ac.jp

Supplementary Table 1: Characteristics of febrile patients with positive blood cultures

Patient	Age	Sex	Symptoms at onset	WBC (/µl)	CRP (mg/dl)	Isolated bacteria by blood culture	Isolated bacteria by catheter culture	Underlying disease
B1	2m	M	fever	8,500	8.51	*Escherichia coli*	not examined	neuroblastoma
B2	1y	F	elevated CRP	500	1.61	*Capnocytophaga sputigena*	not examined	acute lymphoid leukemia
B3	2y	F	fever	<100	1.80	*Streptococcus oralis/mitis*	not examined	neuroblastoma
B4	6y	M	fever	4,500	0.16	*Stenotrophomonas maltophilia*	*Stenotrophomonas maltophilia*	acute lymphoid leukemia
B5	6y	M	fever	<100	9.69	*Staphylococcus aureus*	not examined	neuroblastoma
B6	8y	F	fever, dyspnea	66,000	1.80	*Acinetobacter ursingii*	not examined	acute lymphoid leukemia
B7	8y	M	fever, chills	5,000	0.80	*Stenotrophomonas maltophilia*	*Stenotrophomonas maltophilia*	neuroblastoma
B8	8y	M	fever	<100	0.27	*Streptococcus oralis/mitis*	negative	acute lymphoid leukemia
B9	9y	F	elevated CRP	9,100	3.88	*Bacillus cereus*	*Bacillus cereus*	nasal cavity cancer
B10	10y	M	fever	7,800	0.72	*Sphingomonas paucimobilis*	*Sphingomonas paucimobilis*	neuroblastoma
B11	10y	M	fever, chills	<100	3.88	*Staphylococcus lugdunesis*	not examined	neuroblastoma
B12	10y	F	fever	10,700	15.76	*Staphylococcus epidermidis*	*Staphylococcus epidermidis*	neuroblastoma

WBC, white blood cell count; CRP, C-reactive protein.

Supplementary Table 2: Characteristics of febrile patients with negative blood cultures

Patient	Age	Sex	Symptoms at onset	WBC (/μl)	CRP (mg/dl)	Underling disease
N1	2y	M	fever	1,300	13.46	langerhans histiocytosis
N2	2y	M	fever	14,200	5.55	langerhans histiocytosis
N3	2y	M	fever	15,100	15.72	after liver transplantation
N4	2y	F	fever	1,900	26.23	acute myeloid leukemia
N5	2y	M	fever	11,200	2.40	juvenile myelomonocytic leukemia
N6	2y	M	fever	<100	2.16	yolk sac tumor
N7	3y	F	fever	<100	0.90	neuroblastoma
N8	3y	F	fever	5,100	15.0	sepsis
N9	3y	F	fever	<100	1.82	neuroblastoma
N10	4y	F	fever	<100	8.73	acute lymphoid leukemia
N11	4y	M	fever	<100	6.94	neuroblastoma
N12	6y	M	fever, chills	5,500	0.16	neuroblastoma
N13	7y	F	fever	<100	0.16	neuroblastoma
N14	7y	F	fever, chills	2,400	3.12	acute lymphoid leukemia
N15	8y	F	fever, chills	3,500	8.19	neuroblastoma
N16	9y	F	fever	14,400	14.4	sepsis
N17	10y	M	fever, chills	7,600	0.06	neuroblastoma
N18	10y	M	fever	300	0.40	acute lymphoid leukemia
N19	11y	M	fever	7,300	1.68	aplastic anemia
N20	13y	F	fever, convulsion	3,700	6.82	chronic myelomonocytic leukemia
N21	14y	F	fever	<100	5.23	Wilms' tumor
N22	16y	F	fever	600	17.72	aplastic anemia
N23	17y	M	fever	1,100	0.31	acute myeloid leukemia

WBC, white blood cell count; CRP, C-reactive protein.

Supplementary Table 3: Detection of antimicrobial resistance genes in patients diagnosed by next-generation sequencing

Patient	Detected bacteria by NGS (taxonomic rank)	Detected antimicrobial resistance gene*	Drug susceptibility of cultured bacteria	Drug resistance of cultured bacteria
B1	*Escherichia coli* (species)	strB, dfrA17, aadA5, mph(A), strA	CVA/AMPC, TAZ/PIPC, CMZ, SBT/CPZ, IPM, MEPM, GM, TOB, AMK, MINO, DRPM	ABPC, PIPC, CEZ, CCL, CTX, CTRX, CPDX, CAZ, CFPM, AZT, CPFX, LVFX, ST
B2	*Comamonadaceae* (family)	no genes found	-	-
B3	*Streptococcus* (genus)	mef(A), msr(D)	CTX, CTRX, MEPM, CLDM, LVFX, VCM, CP	EM
B4	*Stenotrophomonas maltophilia* (species)	blaL1, aac(6')-Iz, sph	CAZ, MINO, LVFX, ST	No resistance
B7	*Stenotrophomonas maltophilia* (species)	blaL1, sph	MINO, LVFX, ST	CFPM, CZOP
B9	*Bacillus* (genus)	no genes found	CTRX, IPM, GM, AMK, EM, CLDM, TC, CPFX, VCM, RFP	PCG, ABPC, ST
B10	*Sphingomonadaceae* (family)	no genes found	TAZ/PIPC, CAZ, SBT/CPZ, IPM, MEPM, GM, TOB, AMK, MINO, LVFX, ST	PIPC, AZT
B12	*Staphylococcus epidermidis* (species)	mph(C), blaZ, mecA, dfrG, fosA, msr(A)	GM, CLDM, MINO, LVFX, VCM, TEIC, LZD, FOM, RFP	PCG, ABPC, MPIPC, CEZ, CTM, FMOX, IPM, EM, ST
N1	*Tatlockia micdadei* (species)	aph(3')-IIa, blaTEM-116, blaTEM-157	-	-
N19	*Escherichia coli* (species)	no genes found	-	-

*, Detected antimicrobial resistance gene wad shown, if the fraction of reference covered accounted for more than 50%; ABPC, Ampicillin; AMK, Amikacin; AZT, Aztreonam; CAZ, Ceftazidime; CCL, Cefaclor; CFPM, Cefepime; CEZ, Cefazolin; CLDM, Clindamycin; CPFX, Ciprofloxacin; CPDX, Cefpodoxime; CTX, Cefotaxime; CTRX, Ceftriaxone; CTM, Cefotiam; CVA/AMPC, Clavulanate/Amoxicillin; CZOP, Cefozopran; DRPM, Doripenem; EM, Erythromycin; FMOX, Flomoxef; FOM, Fosfomycin; GM, Gentamicin; IPM, Imipenem; LVFX, Levofloxacin; LZD, Linezolid; MEPM, Meropenem; MPIPC, Methylphenylisoxazolylpenicillin; PCG, Benzylpenicillin; PIPC, Piperacillin; RFP, Rifampicin; ST, Sulfamethoxazole -Trimethoprim; TAZ/PIPC, Tazobactam/Piperacillin; TEIC, Teicoplanin; TOB, Tobramycin; VCM, Vancomycin.

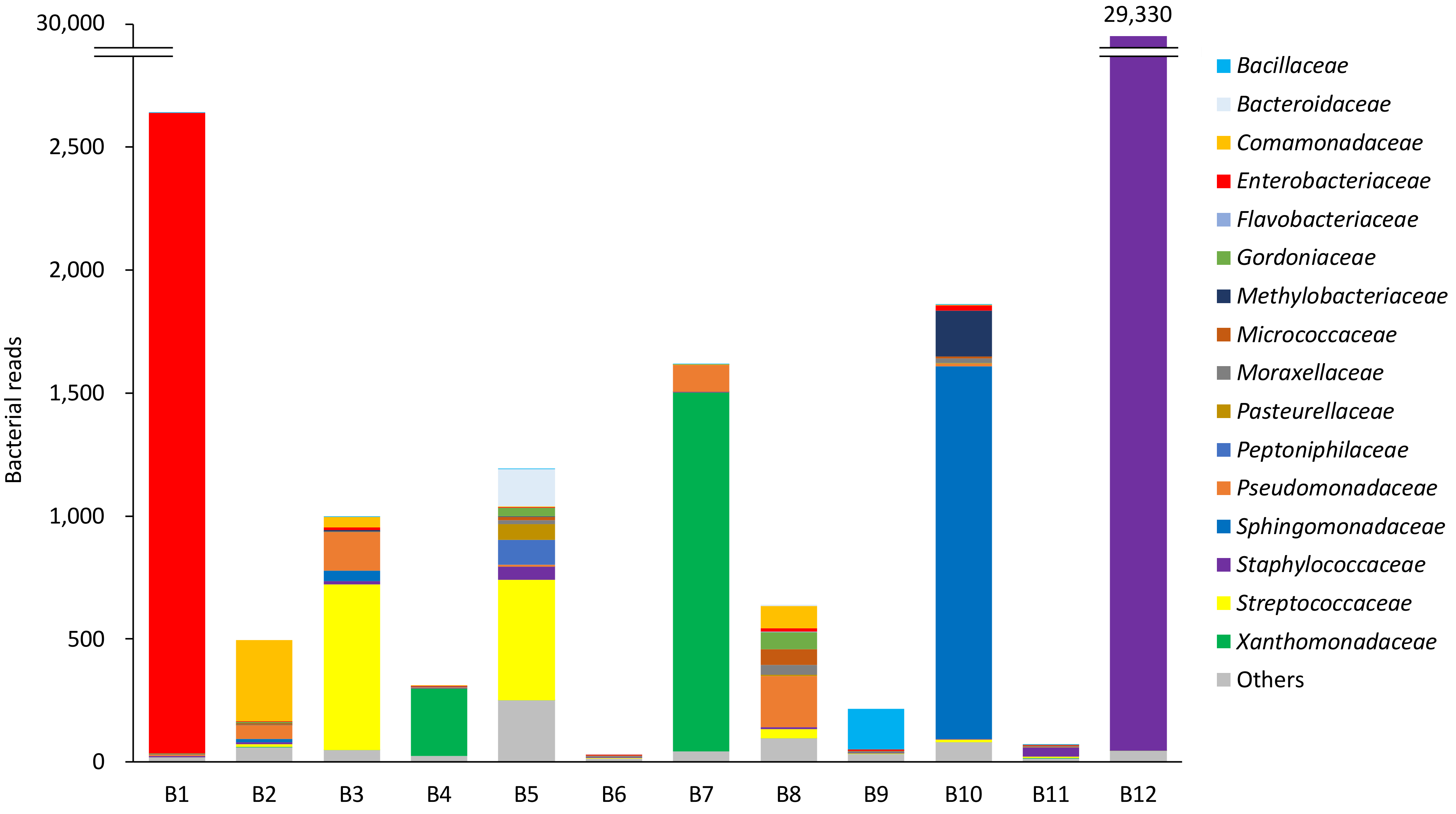

Supplemental Figure 1

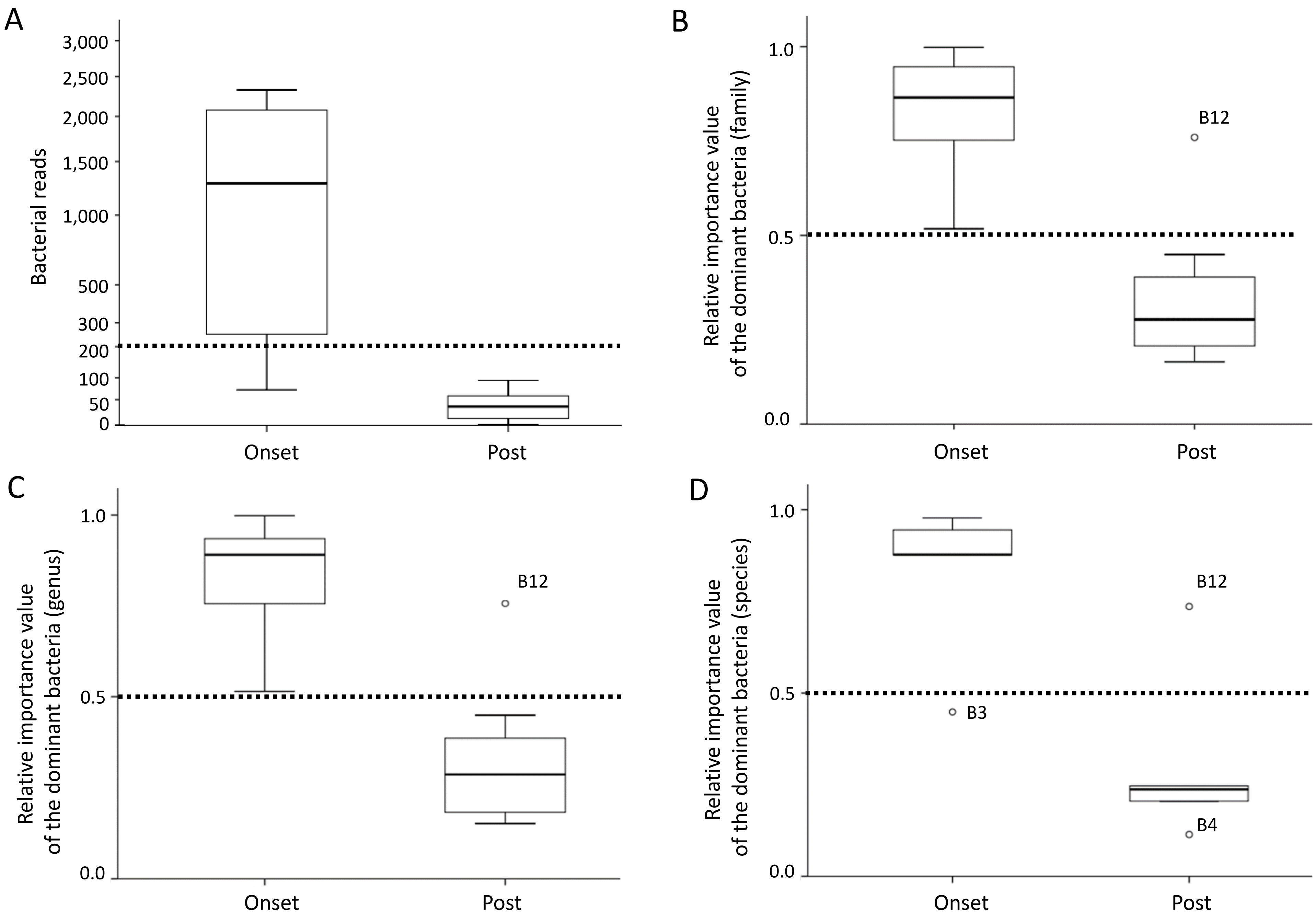

Supplemental Figure 2

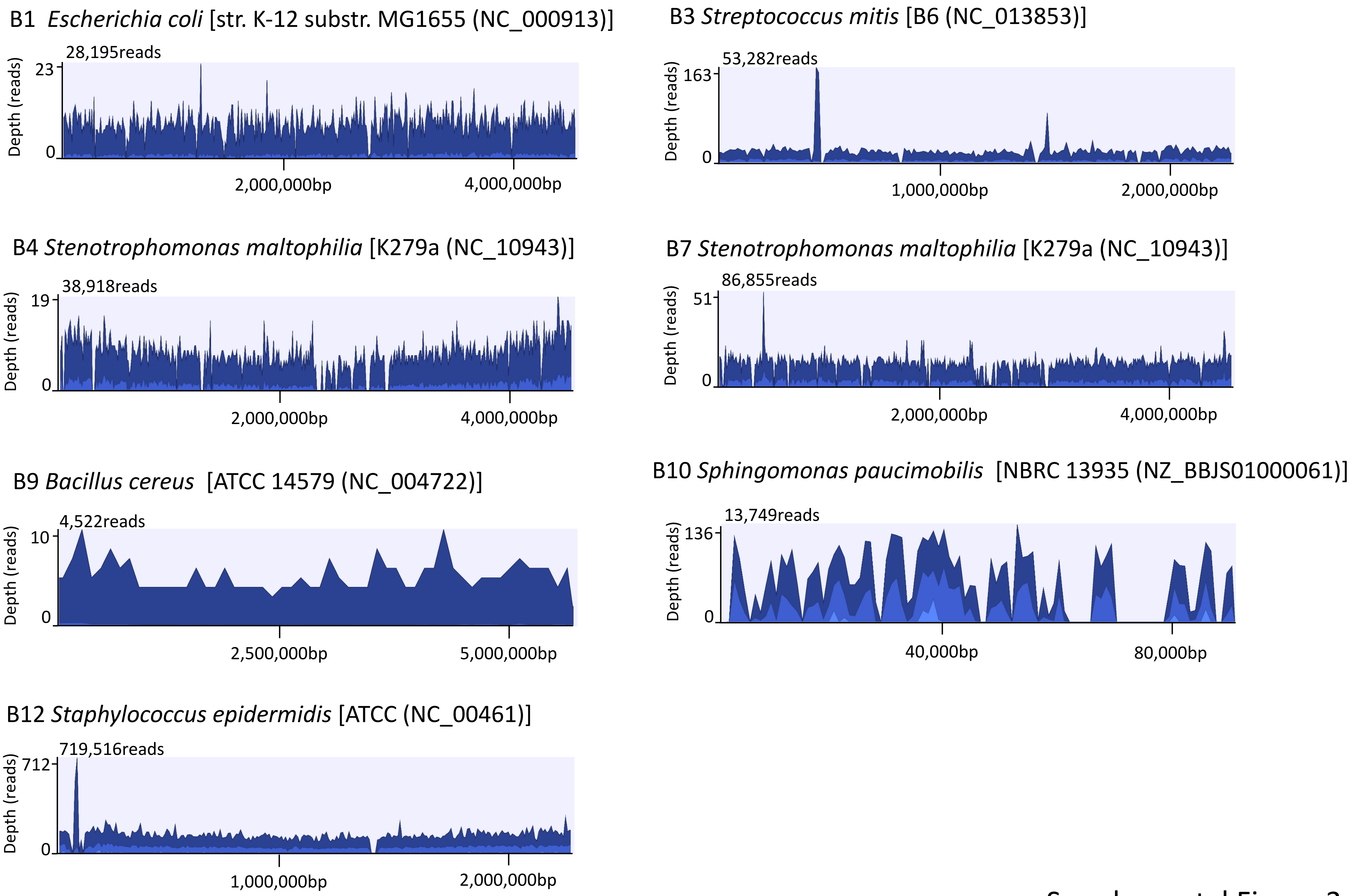

Supplemental Figure 3

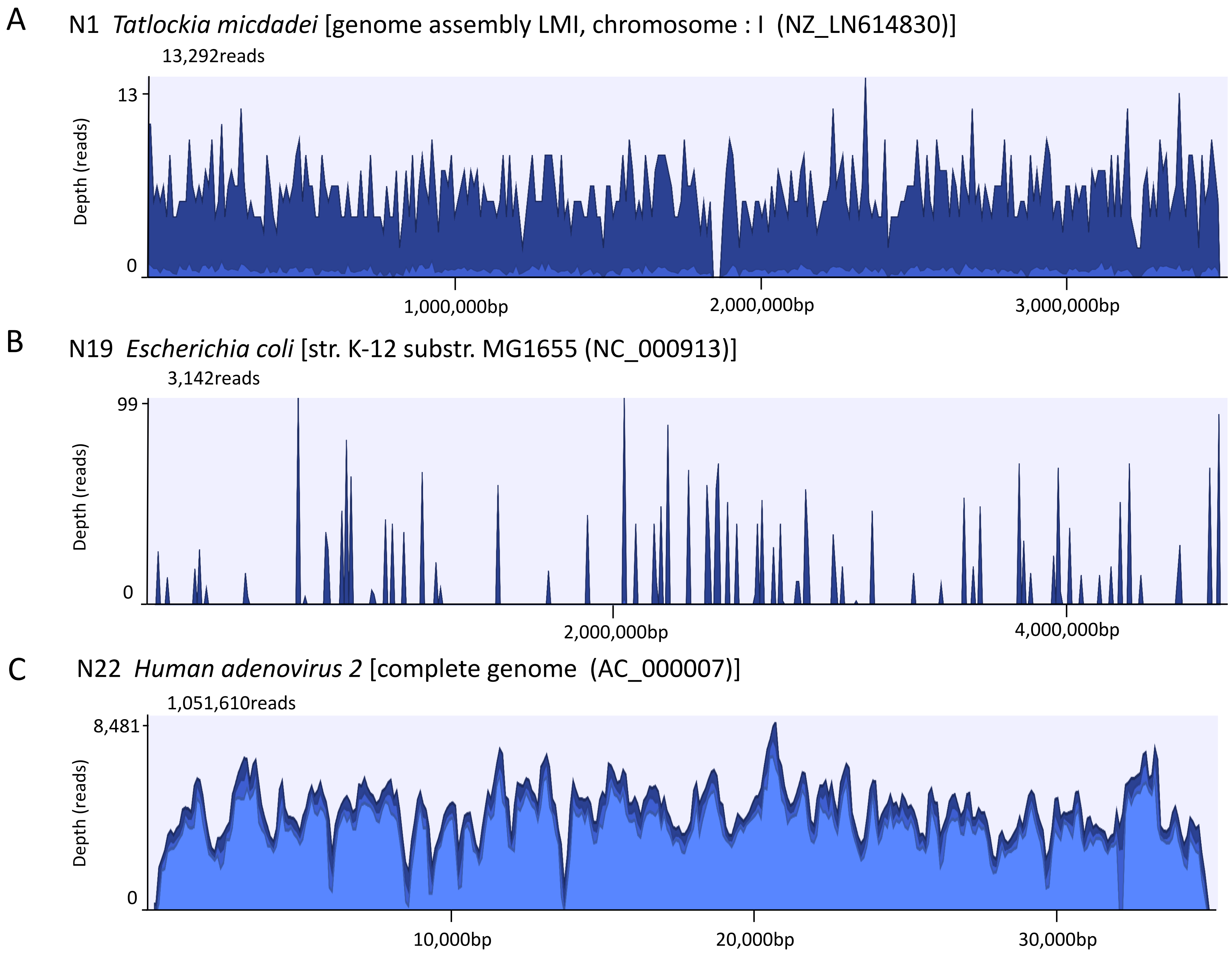

Supplemental Figure 4

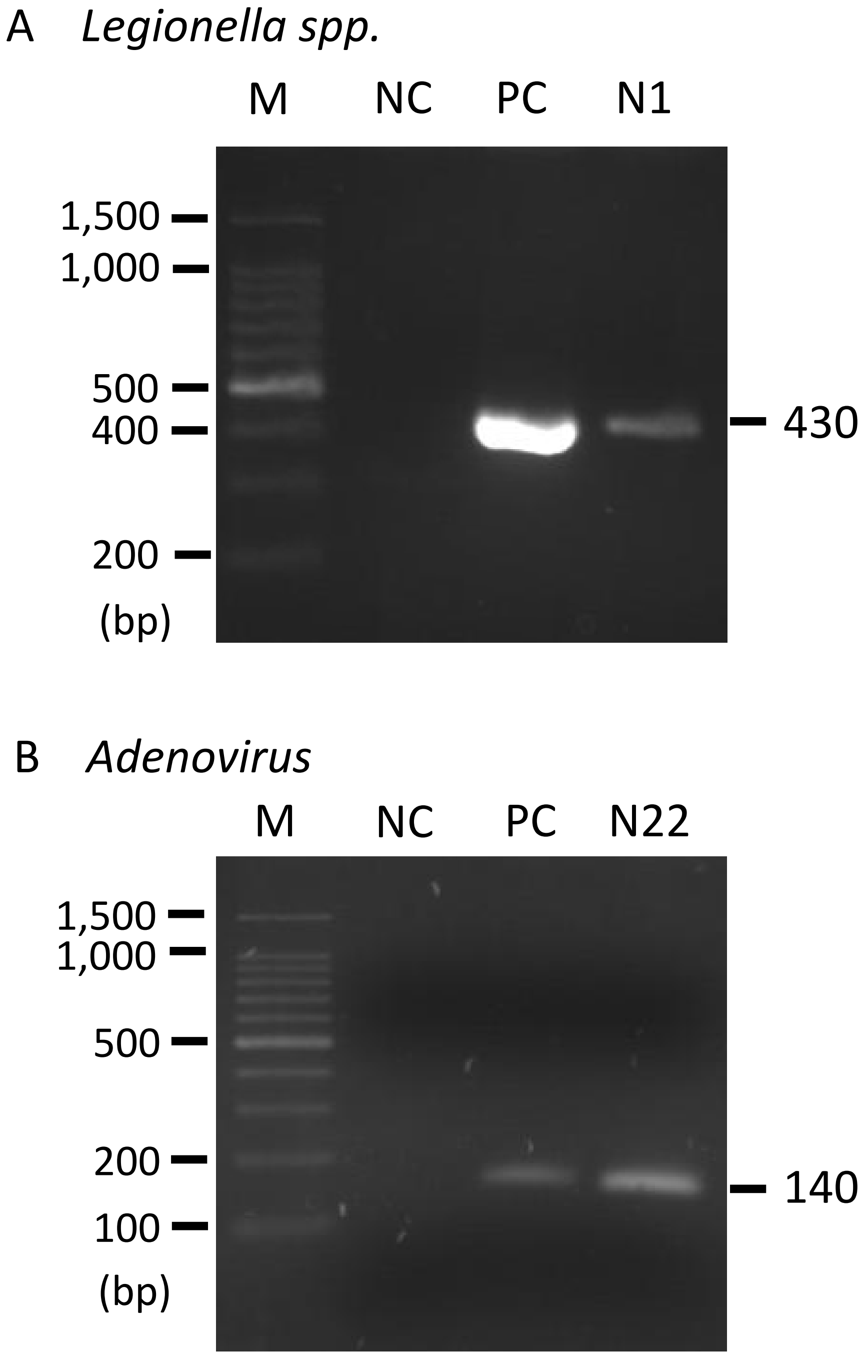

Supplemental Figure 5

Supplementary Figure 1: Bacterial reads per million reads of sequencing in patients with bloodstream infection
Each bar represents taxa at the family level of taxonomic hierarchy. Bacterial reads of patient B12 were more than 10-fold higher than those of the other patients.

Supplementary Figure 2: Bacterial reads per million reads of sequencing and index values in bloodstream infection patients in whom the dominant bacteria by next-generation sequencing was identical to bacteria isolated in blood culture
(A) Bacterial reads per million reads of sequencing (BR) are demonstrated at onset and post-onset of the infection. (B) Relative importance value of the dominant bacteria (P1) at the family level in 8 patients (B1, B3, B4, B7, B9, B10, B11, and B12) is shown at the two time points. (C) P1 at the genus level in 7 patients (B1, B3, B4, B7, B9, B11, and B12) is shown at the two time points. (D) P1 at the species level in 5 patients (B1, B3, B4, B7, and B12) is shown at the two time points.

Supplementary Figure 3: Coverage of reference genomes of the dominant bacteria by next-generation sequencing in patients with bloodstream infection
Sequencing reads were mapped to the reference genomes of the dominant bacteria, which were identical to bacteria isolated in blood culture, in seven patients. Light blue, blue, and dark blue colors in the alignments represent minimal, average, and maximal coverage in aggregated 100-bp regions, respectively.

Supplementary Figure 4: Coverage of reference genomes of causative microorganisms by next-generation sequencing in patients with suspected bloodstream infection
Sequencing reads were mapped to the reference genomes of causative microorganisms in patients N1, N19, and N22. Light blue, blue, and dark blue colors in the alignments represent minimal, average, and maximal coverage in aggregated 100-bp regions in patients N1 and N19 and 10-bp region in patient N22.

Supplementary Figure 5: Amplification of microbial DNA from clinical samples
Legionella spp. and adenovirus were PCR amplified from blood samples from patients N1 and N22, respectively. The expected product size of each PCR is shown. M=100-bp DNA ladder marker, NC=negative control, PC=positive control.
